# Genetics of Alcohol-Induced Behaviors in *Drosophila*

**Published:** 2000

**Authors:** Ulrike Heberlein

**Affiliations:** Ulrike Heberlein, Ph.D., is an associate professor in the Department of Anatomy and the Department of Neurology, Programs in Neuroscience, Developmental Biology, and Biomedical Sciences, University of California at San Francisco, San Francisco, California

**Keywords:** animal model, *Drosophila melanogaster*, AOD sensitivity, cAMP, mutation, adenylate cyclase, genetic trait, animal strains

## Abstract

Researchers frequently study the fruit fly *Drosophila melanogaster* as a model system for mammalian development and behavior. *Drosophila* appear resistant to alcohol’s toxic effects and display many behaviors resembling intoxication (e.g., impaired motor control) when exposed to alcohol vapors. Accordingly, investigators have begun to measure alcohol sensitivity in *Drosophila* and to identify genetic mutations associated with increased or decreased sensitivity. One mutant called *cheapdate* affects a signaling system that plays a role in many regulatory processes in a cell and which involves the compound cyclic adenosine monophosphate (cAMP). Additional *Drosophila* mutants with altered alcohol sensitivity carry mutations in other components of the cAMP signaling system. Because the cAMP system also is affected in human alcoholics, these results indicate that studies using *Drosophila* as a model system may identify genetic changes relevant to human alcoholism.

The fruit fly *Drosophila melanogaster* is one of the most widely used and successful genetic model systems for studying development and behavior. The usefulness of this model system is based on the fact that the genes and biochemical pathways underlying development and behavior have largely been conserved during evolution. As a result, many genes first identified in *Drosophila* have provided major insights into human and other vertebrate development and disease. *Drosophila* has a relatively sophisticated nervous system and is capable of many complex behaviors. For example, the flies can learn to associate certain events and to remember that association (Davis 1996; Dubnau and Tully 1998). Furthermore, they have sophisticated courtship behaviors (O’Dell and Kaiser 1997). Another advantage of *Drosophila* is that they are easy to rear and have a generation time of only approximately 2 weeks, allowing researchers to explore the heritability of certain traits or behaviors over many generations in a short period of time.

Nearly a century of intense genetic research on *Drosophila* has generated innumerable and sophisticated genetic tools, such as chromosomes that carry mutations resulting in visible traits (i.e., phenotypes) that can be used to map novel mutations. Subsequently, such genetic techniques as germ-line transformation^1^ (Rubin and Spradling 1982), use of transposable elements as mutagens (Engels 1983), transposon tagging (Bingham et al. 1981), and targeted gene disruption (Rong and Golic 2000) have allowed researchers to conduct molecular and reverse genetic analyses. Finally, the sequence of the *Drosophila* genome has recently been published, and its analysis has revealed further similarities between flies and mammals (Adams et al. 2000).

*Drosophila* have been used extensively to investigate developmental processes and to study nervous system function. In doing so, researchers have identified many *Drosophila* gene products for whom the corresponding mammalian gene products (i.e., the mammalian homologs) have been implicated as potential targets for alcohol. For example, flies carry homologs of the mammalian receptors for the brain chemicals (i.e., neurotransmitters) gamma-aminobutyric acid, serotonin, dopamine, and glutamate (Littleton and Ganetzky 2000), all of which have been implicated in alcohol’s actions (Tabakoff and Hoffman 1996). Consequently, *Drosophila* may be a suitable animal model to study alcohol’s effects on brain function and alcohol-related behaviors, such as sensitivity and tolerance to alcohol’s effects. This article describes experimental designs to measure alcohol sensitivity and reviews the initial results of alcohol-related genetic research in *Drosophila*.

## Measuring Alcohol Sensitivity in *Drosophila*

The natural habitat of *Drosophila* includes fermenting plants, which often contain high alcohol levels (i.e., 3 or more percent). Accordingly, fruit flies are resistant to alcohol’s toxic effects and can metabolize alcohol efficiently for use as an energy source or as a starting material (i.e., substrate) for the production of lipids (Geer et al. 1993). To test whether a particular *Drosophila* strain is resistant to alcohol’s toxic effects, researchers add alcohol to the culture medium serving as the flies’ food (Geer et al. 1993). Such analyses found that *Drosophila* strains isolated from the wild differ in their resistance to alcohol (Kamping and van Delden 1978; David and Van Herrewege 1993). In addition, researchers found that they could quickly and substantially increase a *Drosophila* population’s resistance to alcohol in the laboratory. For example, resistant strains were obtained by selectively breeding flies that survived exposure to high alcohol levels in their food (Chambers 1991) or were resistant to the effects of alcohol vapor (Cohan and Hoffman 1986; Weber and Diggins 1990).

When exposed to alcohol vapor, adult *Drosophila* display many behaviors resembling acute intoxication in mammals, such as impaired motor control. To measure alcohol’s effects on locomotion, researchers place flies into a small chamber that is covered with grid lines. Locomotor behavior is measured by counting the number of lines of the grid crossed as a function of time (Bainton et al. 2000; Singh and Heberlein in press). Within a few minutes of exposure, the flies become hyperactive and disoriented and then uncoordinated and sedated. After approximately 20 minutes of exposure, they become immobile, but recover 5 to 10 minutes after the alcohol is withdrawn (Singh and Heberlein in press).

Studies found that in rodents, alcohol’s locomotor-stimulating effects are modulated by nerve-cell systems using the neurotransmitter dopamine (i.e., dopaminergic systems) (Phillips and Shen 1996). To explore a potential role for dopamine in alcohol responses in *Drosophila*, researchers tested flies with severely reduced dopamine levels for alcohol-induced changes in locomotor behavior using the test described above. In these flies, alcohol’s ability to induce locomotor activation was significantly reduced; however, alcohol-induced sedation was normal (Bainton et al. 2000). These data suggest that dopaminergic systems play a similar role in the responses of rodents and *Drosophila* to acute alcohol exposure.

Another approach to measuring alcohol’s effects on *Drosophila* is to determine their inability to stand (i.e., loss of postural control) after alcohol intake using an inebriometer. This instrument, which was designed originally to selectively breed alcohol-resistant flies (Weber and Diggins 1990), consists of a 125-cm long vertical tube containing a series of sloping mesh baffles on which flies can stand (see figure). The flies are placed at the top of the tube. Then, alcohol vapor is circulated through the tube, causing the flies to become intoxicated. As a result, the flies lose postural control and begin to fall through the tube. Their sensitivity to alcohol intoxication is measured by the time required for them to emerge from the bottom of the tube at a particular alcohol concentration. Thus, flies that are more sensitive to alcohol-induced loss of postural control emerge from the tube after a shorter time than do flies that are more resistant to alcohol’s effects.

## Genetic Analyses of Alcohol-Related Traits

Researchers have conducted genetic analyses in *Drosophila* to identify mutations that alter sensitivity to alcohol intoxication (Singh and Heberlein in press). To this end, flies were treated with a chemical (i.e., ethyl methane sulfonate) that induces lesions in the DNA. The researchers then tested many thousands of potentially mutant flies in the inebriometer to identify those strains that exhibited altered alcohol-induced behaviors. This approach identified mutants that displayed either increased or decreased sensitivity to a single alcohol exposure (Singh and Heberlein in press).

Two of these mutant strains were named *tipsy* and *barfly* to reflect their increased and reduced sensitivity to alcohol, respectively. Both these mutant strains responded normally when tested for alcohol-induced locomotor activation. However, *tipsy* flies became sedated at alcohol concentrations that were lower than those needed to sedate normal (i.e., wild-type) flies. Conversely, *barfly* mutants required higher alcohol doses than did wild-type flies to achieve sedation (Singh and Heberlein in press). These results suggest that the genes that control alcohol’s activating effects can differ from the genes regulating alcohol’s sedative effects. Analyses of well-established rodent models for alcohol-induced behaviors have led to similar conclusions (Shen et al. 1996). The identification of the genes disrupted by these mutations in flies should help shed light onto the mechanisms by which acute alcohol exposure modulates behavior.

An alternative approach to generate mutations in *Drosophila* involves the use of a transposable element (i.e., a short piece of DNA named P-element) that integrates randomly into the flies’ DNA and inactivates any genes located near the integration site (Engels 1983). One mutant generated using this method that shows increased sensitivity to alcohol was called *cheapdate* (Moore et al. 1998). Further analysis demonstrated that *cheapdate* flies carry a mutation in a gene called *amnesiac*, which was identified originally because of its role in learning and memory (Quinn et al. 1979). The *amnesiac* gene is believed to encode a neuropeptide that shows some similarity to a vertebrate peptide that activates the enzyme adenylate cyclase (AC) (Feany and Quinn 1995). AC promotes the formation of a compound called cyclic adenosine monophosphate (cAMP), which is involved in numerous regulatory and signaling pathways in the cell.

In addition to the *cheapdate* mutant, other findings from *Drosophila* studies indicate that the cAMP signaling system appears to play a role in mediating alcohol’s effects. Thus, *Drosophila* mutants with disruptions in the gene encoding a certain type of AC also display increased sensitivity to alcohol (Moore et al. 1998). Furthermore, researchers recently reported that a *Drosophila* strain carrying a mutation in a gene that encodes part of an enzyme called cAMP-regulated protein kinase (PKA–RII) is more resistant to the acute effects of alcohol than are wild-type flies (Park et al. 2000). Interestingly, mice carrying a mutation in the homologous PKA–RII gene also show increased alcohol resistance and voluntarily consume greater amounts of alcohol (Thiele et al. 2000).

Taken together, these findings indicate that mutations that disrupt the flies’ ability to properly regulate cAMP levels also affect their sensitivity to alcohol. This observation is particularly interesting, because studies in humans found that alcoholics exhibit reduced levels of AC activity (Diamond et al. 1987; Tabakoff et al. 1988) and that the cAMP signaling pathway is sensitive to alcohol’s effects (Diamond and Gordon 1997). Finally, the finding that disruption of a single gene (i.e., PKA–RII) leads to similar alterations in the alcohol responses of mice and flies provides provocative evidence for evolutionary conservation of the underlying molecular mechanisms.

## Conclusions

The particular forward genetic approach described in this article can potentially result in the isolation of all genes that influence alcohol-related behaviors. Therefore, this approach is a powerful means of obtaining unbiased information about the mechanisms underlying those behaviors. Initial analyses suggest that this approach can indeed identify genes contributing to alcohol-related biochemical phenotypes (e.g., genes influencing the AC pathway) and, therefore, is viable and relevant. However, it is too soon to speculate whether these genes will provide clues to alcohol addiction in humans.

The identification of *Drosophila* genes that disrupt alcohol-related behaviors provides at least two potential benefits. First, identifying these genes could facilitate studies in vertebrates, particularly humans, because the vertebrate homologs could be investigated as potential candidate genes. Second, such analyses could provide researchers with tools to investigate in *Drosophila* the mechanisms underlying alcohol-induced behaviors in higher organisms. For example, identifying novel genes associated with alcohol-related phenotypes in mammals or humans may provide few clues for the development of therapy because of the difficulties of studying these genes in complex mammalian systems. The introduction of such genes into *Drosophila*, however, might allow researchers to identify the mechanism through which the novel gene acts in a simpler model system.

## Figures and Tables

**Figure:**
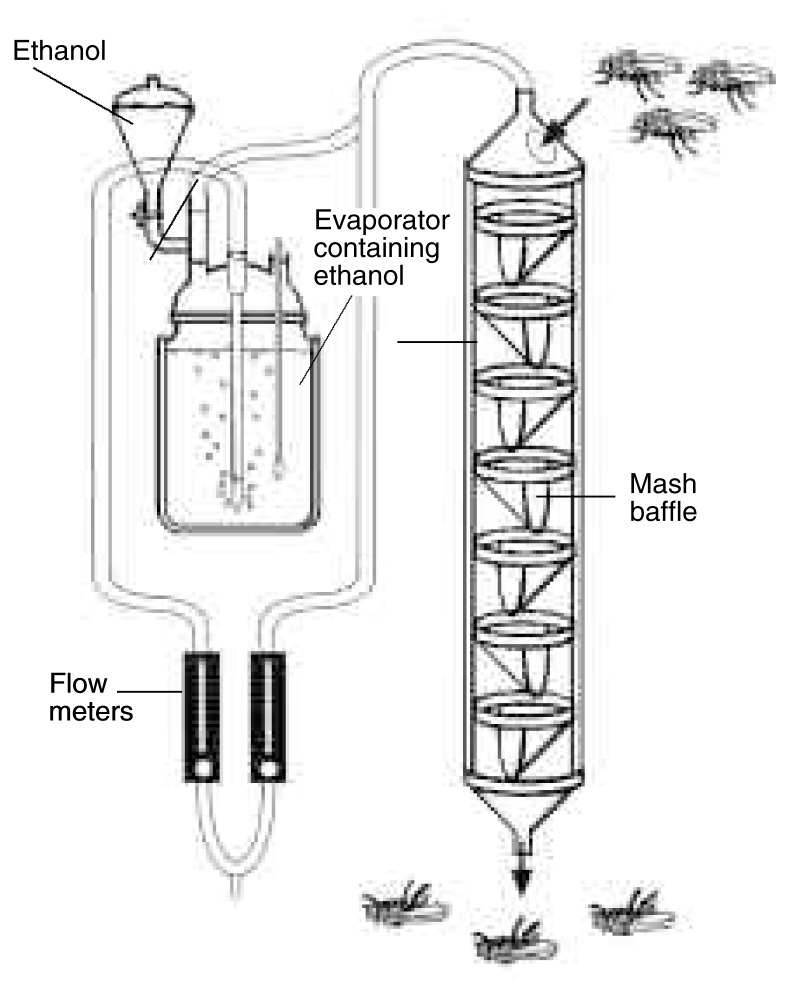
The inebriometer is an apparatus that is used to measure the sensitivity of *Drosophila* to alcohol vapor. Approximately 100 flies are introduced into the top of a 4-foot glass column through which a controlled concentration of alcohol vapor circulates. As they become intoxicated, the flies progressively lose postural control and tumble downwards; their fall is impeded by their ability to cling to oblique mesh baffles distributed along the length of the column. The time required for the flies to emerge at the bottom of the column is a measure of their alcohol sensitivity.

## Footnotes

1 For a definition of this and other technical terms used in this article, please see the central glossary, pp. 193–195.
